# Editorial: The pharmacological effects and mechanisms of drugs against human diseases by modulating redox homeostasis

**DOI:** 10.3389/fphar.2023.1200137

**Published:** 2023-07-14

**Authors:** Ying Zhang, Luchen Shan, Fuchun Yang, Zhihao Liu, Ming Xu

**Affiliations:** ^1^ Graduate School of Medicine, Yamaguchi University, Yamaguchi, Japan; ^2^ International Cooperative Laboratory of Traditional Chinese Medicine Modernization and Innovative Drug Development of Chinese Ministry of Education, Institute of New Drug Research, Jinan University College of Pharmacy, Guangzhou, China; ^3^ Department of Cancer Biology, University of Cincinnati College of Medicine, Cincinnati, OH, United States; ^4^ Department of Clinical Pharmacology, College of Pharmacy, Dalian Medical University, Dalian, China

**Keywords:** herbal medicine, redox homeostasis, pharmacological effects, reactive oxygen species, bioactive molecule

Redox homeostasis is a critical process in maintaining proper cell, tissue, and organ functions in the body. In the past decades, research in the field of redox homeostasis has become increasingly popular. With the advancement of science, two new concepts of oxidative eustress and oxidative distress have been proposed (Niki, 2016). Physiologically, a complex network of antioxidant enzymes, including superoxide dismutase, catalase, and glutathione peroxidase, plays a critical role in maintaining redox homeostasis by neutralizing ROS and preventing oxidative damage, denoted as oxidative eustress (Sies, 2021). On the other hand, oxidative distress which means supraphysiological oxidative challenges/damages are associated with multiple disorders, such as neurodegenerative diseases including Alzheimer’s disease, Parkinson’s disease, Huntington’s disease (Korovesis et al., 2023); ischemia diseases (Chen and Li, 2020); cancers (Lyons et al., 2023); chronic inflammatory disorders (IBD) (Alemany-Cosme et al., 2021); and metabolic disorders such as diabetes and NAFLD (Braud et al., 2017; Rendra et al., 2019). Interestingly, some ROS, such as H_2_O_2_, as important cell signaling molecules may also help explain the rather limited clinical success of antioxidants (Forman and Zhang, 2021). Research on the pathogenic mechanisms of ROS and development of ROS-modulating drugs has been become a popular field of study in recent decades. This special Research Topic of articles is dedicated to “The pharmacological effects and mechanisms of drugs against human diseases by modulating redox homeostasis.” The goal is to cover the latest research on ROS signaling pathways in diseases development and drugs to treat diseases by modulating ROS levels.

A total of eight basic research articles and literature reviews have been published in this Research Topic. These articles cover a variety of drugs used to combat diseases by modulating redox homeostasis.

To continue, four articles report the role of antioxidant drugs in digestive diseases. In nonalcoholic fatty liver disease (NAFLD), a chronic advanced liver disease, ROS production increases due to the accumulation of free fatty acids in the liver (Delli Bovi et al., 2021). Thus, ROS plays a crucial role in the progression of NAFLD. Moreover, ROS can cause lipid peroxidation, which leads to the accumulation of toxic lipid metabolites, and in turn damages cellular membranes and organelles. Interventions to target ROS production and oxidative stress may be promising strategies for preventing and treating NAFLD. Ding et al. reported that epigallocatechin gallate (EGCG) can alleviate liver injury, lipid accumulation, oxidative stress, hepatic steatosis, and decrease iron overload, while also inhibiting ferroptosis in a murine model of NAFLD. The authors further demonstrated that EGCG exerts protective effects against hepatic lipotoxicity by inhibiting mitochondrial ROS-mediated hepatic ferroptosis. This provided a new perspective on potential prevention and treatment strategies for NAFLD. Wu et al. investigated the effect of bicyclol, a clinical medicine, on NAFLD. The authors conducted proteomics analyses and validation experiments, which confirmed the therapeutic effect of bicyclol on NAFLD, revealing that this effect may be linked to signaling pathways associated with bile acid metabolism, cytochrome P450-mediated metabolism, metal ion metabolism, angiogenesis, and immunological responses. On the other hand, ROS has been implicated in the pathogenesis of many intestinal diseases, including IBD, colorectal cancer, and intestinal ischemia-reperfusion (II/R) injury. Li et al. discovered that II/R could induce an inflammatory response and oxidative stress, which subsequently activated the NOD-, LRR-, and pyrin domain-containing 3 (NLRP3) inflammasome and caused damage to intestinal and lung tissue. However, these pathological effects were significantly attenuated by Corilagin (Cor). The authors further demonstrated that Cor may inhibit ROS-induced NLRP3 inflammasome activation and pyroptosis both *in vivo* and *in vitro*, suggesting the potential therapeutic benefits of Cor in mitigating II/R-induced tissue injury. Arenbaoligao et al. demonstrated that Kumatakenin, a Chinese medicine, can protect against IBD-induced epithelial ferroptosis injury in colonic tissues. Using RNA sequencing and molecular docking methods, the authors verified that Kumatakenin suppresses IBD-induced epithelial ferroptosis by modulating the Eno3-iron regulatory protein axis. These findings provide a scientific rationale for the clinical application of Kumatakenin in the treatment of colitis.

Apart from treating diseases through antioxidant signal pathways, other drugs were designed to increase ROS levels in specific parts of the body, such as anti-cancer, anti-*Escherichia coli* (*E. coli*) bacteria and protective autophagy. Several studies have reported that antioxidant approaches can indeed be used to prevent carcinogenesis (Amstad et al., 1990; Ray and Husain, 2002). On the other hand, since cancer cells are more sensitive to oxidative stress than normal cells, some ROS-inducing chemicals are used to treat established tumors (Wang et al., 2021). Li et al. reported on the anti-cancer effect of *Sanguisorba officinalis* L. (SOL). The authors demonstrated that SOL could increase ROS levels and inhibit the proliferation, migration, and invasion of non-small cell lung cancer cells. Ebselen, a glutathione peroxidase mimic has been confirmed to reduce ROS levels by neutralizing H_2_O_2_ (Nakamura et al., 2002). However, Ebselen can also increase ROS level with a high concentration in yeast cells (Azad et al., 2014). In this Research Topic, Chen et al. reported on the synergistic anti-bacterial effect of the combination of SBC3 and Ebselen against *E. coli* and other difficult-to-treat Gram-negative bacteria, achieved by increasing ROS levels through the ROS pathway. These two articles provide new perspectives on the potential use of drugs for disease treatment by regulating ROS levels.

In summary, this Research Topic focused on the regulation of oxidative homeostasis as a potential strategy for treating diseases. The information gained from this Research Topic may help identify new biomolecules for disease treatment and drive the development of novel therapeutic drugs.
